# Colorectal cancer under 20 years old: a retrospective analysis from three tertiary hospitals

**DOI:** 10.1007/s00432-020-03397-2

**Published:** 2020-09-23

**Authors:** Chengjing Zhou, Weiwei Xiao, Xiaohao Wang, Haiyang Chen, Shaoqing Niu, Qiaoxuan Wang, Hui Chang, Xiaojun Wu, Peirong Ding, Zhizhong Pan, Xiangbo Wan, Yong Bao, Yuanhong Gao

**Affiliations:** 1 Department of Radiation Oncology, State Key Laboratory of Oncology in South China, Collaborative Innovation Center for Cancer Medicine, Sun Yat-Sen University Cancer Center, 651 Dongfeng Road East, Guangzhou, 510060 China; 2 Department of Radiation Oncology, The Sixth Affiliated Hospital of Sun Yat-Sen University, Guangzhou, 510655 China; 3 Department of Radiation Oncology, The First Affiliated Hospital of Sun Yat-Sen University, Guangzhou, 510080 China; 4 Department of Colorectal Surgery, State Key Laboratory of Oncology in South China, Collaborative Innovation Center for Cancer Medicine, Sun Yat-Sen University Cancer Center, Guangzhou, 510060 China

**Keywords:** Colorectal cancer, Pediatric cancer, DNA mismatch repair, Therapy

## Abstract

**Purpose:**

Colorectal cancer (CRC) rarely occurs in children and adolescents. This study aimed to perform a retrospective analysis and disclose more detailed information about CRC in patients under 20 years old.

**Methods:**

Medical records of CRCs in patients under 20 years old referred to three tertiary hospitals in China from September 2000 to July 2019 were retrospectively reviewed. Clinicopathological characteristics, treatment processes and laboratory findings were summarized and treatment outcomes and prognostic factors were analyzed.

**Results:**

A total of 33,394 CRC medical records were analyzed, and we identified seventy (0.21%) CRCs in patients under 20. The most common primary tumor location was the left hemicolon (35.7%). The prominent pathological types were mucinous adenocarcinoma (22.9%) and signet ring cell carcinoma (22.9%). Nearly half (47.1%) of the patients presented with distant metastasis at diagnosis. The fractions of patients with deficient mismatch repair (dMMR) protein expression and microsatellite instability-high (MSI-H) were 23.8% (5/21) and 71.4% (5/7), respectively. Forty-four patients underwent radical surgery. Fifty-five patients received chemotherapy and six patients received radiotherapy. One dMMR/MSI-H rectal cancer patient received immunotherapy and achieved a clinically complete response. The median overall survival (OS) time was 80 months. The 3-year and 5-year OS rates were 61.8% and 57.2%, respectively. An absence of distant metastasis was a favorable factor for OS. For stage II/III CRCs, classic adenocarcinoma and radical surgery were favorable factors for OS. For stage IV CRCs, primary location at the colon was a favorable factor for OS.

**Conclusion:**

Child and adolescent CRC patients are likely to have distant metastasis, undifferentiated, left hemicolon location, and a dMMR/MSI-H phenotype at diagnosis. Additional efforts are needed to improve their survival outcomes.

## Introduction

Colorectal cancer (CRC) is the third most common cancer in incidence and ranks second in terms of cancer-related mortality (Bray et al. 2018). Adolescent and young adult (AYA) cancers accounted for 5% of all newly diagnosed invasive cancers in the United States between 2011 and 2015 (Close et al. 2019). We have witnessed an overall trend of decreased incidence of CRC over the past few decades as a result of using colonoscopy and other screening modalities in the older population (Connell et al. 2017). However, CRC incidence in young individuals is steadily rising (Kasi et al. 2019). AYA CRCs present with more advanced stage, poorer cell differentiation, and higher prevalence of signet ring cell histology, and the primary tumors are commonly located in the left side of the colon at diagnosis. Although CRC accounted for only 2.5–3.5% of cancer incidence in adolescents from 15 to 19 years old from 2011 to 2015, the 5-year survival rate was lower than in many other cancers (Close et al. 2019). Both the incidence and mortality rates of CRC are increasing in China in recent decades (Arnold et al. 2017). There are few reports on child and adolescent CRCs in patients under 20 (Kaplan et al. 2019; Indini et al. 2017; Sultan et al. 2010; Yang et al. 2010), especially in China (Du et al. 2015). To better characterize the clinical features, treatment strategies, and outcomes of CRC in children and adolescents, we performed an analysis of CRCs from three tertiary hospitals in South China.

## Materials and methods

### Patient population

Patients with pathologically diagnosed CRC in three tertiary hospitals (Sun Yat-sen University Cancer Center, the First Affiliated Hospital, and the Sixth Affiliated Hospital of Sun Yat-sen University, Guangzhou, China) from September 2000 to July 2019 were initially considered. Eligibility criteria included the following characteristics: (1) pathologically diagnosed classic adenocarcinoma, mucinous adenocarcinoma (MA), or signet ring cell carcinoma (SRCC); and (2) under 20 years of age. Patients were excluded using following criteria: (1) prior history of other malignancies; and/or (2) severe hematopoietic, heart, lung, liver, or kidney dysfunction. Clinical characteristics, treatment process, laboratory findings, and survival outcomes were captured from the medical records and follow-up systems.

### Ethics

The present study was performed in accordance with the ethical standards as presented in the 1964 Declaration of Helsinki and its later amendments or comparable ethical standards and was approved by the Ethics Committee of Sun Yat-sen University Cancer Center with a waiver of informed consent because this research was retrospective and did not involve accessing any patient identification data.

### Tumor location

The colon comprises the left hemicolon, the right hemicolon, and the transverse colon. The left hemicolon includes the left flexura, the descending colon, and the sigmoid, and the right hemicolon comprises the cecum, the ascending colon, and the right flexura. The rectum compromises the anus to 15 cm above the anocutaneous line.

### Staging and tumor biomarkers

All tumors were staged or restaged according to the 8th edition of the American Joint Committee on Cancer (AJCC) TNM staging system. Carcinoembryonic antigen (CEA) and carbohydrate antigen 19–9 (CA19-9) were assessed at diagnosis or when patients were referred to one of the above three hospitals.

### Tumor molecular characterization

Primary tumors were used for tumor molecular analysis. Mismatch repair (MMR) proteins, including MLH1, MSH2, MSH6, and PMS2, were assessed by immunohistochemistry (IHC). Tumors were classified as MMR-deficient (dMMR) if loss of one or more of the proteins was shown. Microsatellite status was detected by next-generation sequencing (NGS) or polymerase chain reaction (PCR) of five microsatellite markers, including BAT25, BAT26, D5S346, D2S123, and D17S250. MSI-high (MSI-H) was defined as presence of ≥ 30% mutations as detected by NGS or ≥ 2 microsatellite marker instability by PCR. K-ras mutation was assessed by NGS and/or PCR.

### Follow-up

All patients were followed at 3-month intervals during the first 2 years, at least every 6 months thereafter for an additional period of 3 years, and then once a year until March 2020. Overall survival (OS) was defined from the date of diagnosis until death from any cause or was censored at last follow-up.

### Statistical analysis

Continuous data are presented as the median with range, and categorical data are presented as proportions (%). Proportions were compared using a *χ*^2^ test. Survival rates were compared with the log-rank test. A two-sided *p* value of < 0.05 was considered statistically significant. Covariates with *p* value < 0.05 by univariate analysis were subjected to multivariate analysis. All statistical analyses were performed using IBM SPSS Statistics 25.0 (IBM Co, Armork, NY, USA) and GraphPad Prism version 8.3.0 (GraphPad Software, Inc., La Jolla, CA, USA).

## Results

### Patient characteristics

A total of 33,394 CRC medical records were analyzed, and we identified 70 CRCs (0.21%) in patients who were under 20 years old with histological diagnoses of adenocarcinoma, some of whom were referred from other hospitals. The characteristics of the patients are shown in Table 1. Of the 70 patients, 28 were diagnosed from September 2000 to December 2009, and the other 42 patients were diagnosed between January 2010 and July 2019. The median age at diagnosis was 18 (range, 8–20), with 30 patients (42.9%) being less than 18 years old. The proportion of male and female patients was 60.0% and 40.0%, respectively. Eleven patients (15.7%) had a family history of malignant tumor, and only 3 (4.3%) had a family history of CRC (one was a parent).

**Table 1:** Clinicopathological characteristics of adolescent colorectal cancer (*n* = 70)

Characteristics	*n*	%
Age (years)		
< 18	30	42.9
18–20	40	57.1
Gender		
Male	42	60.0
Female	28	40.0
Symptoms		
Abdominal pain	33	47.1
Hemafecia	22	31.4
Diarrhea	6	8.6
Abdominal distension	5	7.1
Acute intestinal obstruction	3	4.3
Abdominal mass	2	2.9
Other symptoms	5	7.1
Tumor stage		
II	9	12.9
III	25	35.7
IV	33	47.1
Unknown^a^	3	4.3
Lymph node metastasis		
Positive	41	58.6
Negative	10	14.3
Unknown	19	27.1
Pathological classification		
Mucinous adenocarcinoma	16	22.9
Signet ring cell carcinoma	16	22.9
Classic adenocarcinoma	38	54.3
Primary tumor site		
Colon	50	71.4
Right hemicolon	16	22.9
Transverse colon	8	11.4
Left hemicolon	25	35.7
Unknown	1	1.4
Rectum	19	27.1
Multiple sites	1	1.4
Family history		
Colorectal cancer	3	4.3
Other cancers	8	11.4
None	59	84.3
CEA (ng/ml)^b^		
≤ 5	17	37.0
> 5	29	63.0
CA19-9 (U/ml)^c^		
≤ 35	27	58.7
> 35	19	41.3
MMR status^d^		
dMMR	5	23.8
pMMR	16	76.2
Microsatellite status^e^		
MSI-H	5	71.4
MSS	2	28.6
K-ras^f^		
Mutation	7	63.6
Wild type	4	36.4
Surgery		
Palliative	16	22.9
None	10	14.3
Radical	44	62.9
Chemotherapy		
Yes	56	80.0
No	14	20.0
Radiotherapy		
Yes	6	8.6
No	63	91.4

*MMR* mismatch repair, *dMMR* deficient of mismatch repair, *pMMR* proficient of mismatch repair, *MSI-H* microsatellite instability-high, *MSS* microsatellite stability, *CEA* carcinoembryonic antigen, *CA19-9* carbohydrate antigen 19–9

^a^Stage in two patients was II or III

^b^Data of 46 patients were available

^c^Data of 46 patients were available

^d^Data of 21 patients were available

^e^Data of 7 patients were available

^f^Data of 11 patients were available

### Symptoms and presentation

Six (8.6%) patients presented with multiple symptoms. Common symptoms included abdominal pain (33/70, 47.1%), hemafecia (22/70, 28.6%), diarrhea (6/70, 8.6%), and abdominal distention (5/70, 7.1%). Three patients complained of acute intestinal obstruction and two patients felt an abdominal mass themselves. One patient presented with altered bowel habits, one patient had weight loss, and one patient was diagnosed incidentally during examination for his left leg pain (see Table 1).

### Tumor location

The most common primary tumor location was the left hemicolon (25/70, 35.7%), followed by the rectum (19/70, 27.1%), right hemicolon (16/70, 22.9%), and transverse colon (8/70, 11.4%) (see Table 1). Synchronous multiple primary tumors of the cecum and sigmoid were only confirmed in one patient.

### Pathology and staging

Classic adenocarcinoma, MA and SRCC were diagnosed in 38 patients (54.3%), 16 patients (22.9%), and 16 patients (22.9%), respectively. Almost all MAs and SRCCs were in the colon except four rectal MAs and one rectal SRCC. Thirty-three patients (47.1%) presented with distant metastasis in the abdominal and pelvic cavity (*n* = 16), liver parenchyma (*n* = 9), peritoneal surface (*n* = 8), lung (*n* = 3), distant lymph nodes (*n* = 3), bone (*n* = 2), and ovarian parenchyma (*n* = 1). Regional lymph node status was available in 51 patients, 41 of whom had positive regional lymph nodes. None of the patient presented with stage I disease. The number of patients with stage II and III disease was 9 and 25, respectively. Staging in 3 patients was unclear, but 2 of them were stage II or III. There were 36 patients (51.4%) with locally advanced CRC (LACRC) and 33 patients (47.1%) with distant metastasis (see Table 1).

### Laboratory and molecular tests

Among 46 patients with available serum CEA and CA19-9 data, 29 patients (63.0%) exhibited elevated CEA (> 5 ng/ml) and 19 patients (41.3%) exhibited elevated CA19-9 (> 35 U/ml) levels (see Table 1).

Eleven patients exhibited K-ras mutations, and the proportion of K-ras mutation was 54.6% (6/11) (see Table 1).

MMR proteins were examined in 21 patients. Five patients (23.8%) exhibited dMMR. MLH1 and PMS2 were both negative in two rectal cancer patients, MSH2 and MSH6 were both negative in another rectal cancer patient and a left hemicolon cancer patient, and PMS2 and MSH2 were both negative in one patient with transverse colon cancer.

Microsatellite status was assessed in seven patients. Five patients exhibited MSI-H, three of whom had rectal cancer and two of whom had left hemicolon cancer. Microsatellite stability (MSS) was found in one right hemicolon cancer patient and one transverse colon cancer patient.

Five patients were detected with both MMR and MSI. Two were dMMR and MSI-H; one was pMMR and MSS. MMR and MSI status was inconsistent in two patients. One was pMMR but MSI-H, and the other was dMMR but MSS. Detailed MMR and MSI information in these five patients is shown in Table 2.

**Table 2:** Detailed MMR and MSI information for five patients

Patient no.	Location	Deficient MMR protein	dMMR	MSI test way	Positive microsatellite locus	MSI-H
1	Rectum	MLH1 and PMS2	Yes	PCR	BAT25, BAT26 and D5S346	Yes
2	Rectum	MSH2 and MSH6	Yes	PCR	BAT26 and D5S346	Yes
3	Left hemicolon	No	No	PCR	BAT25 and BAT26	Yes
4	Transverse colon	PMS2 and MSH2	Yes	PCR	No	No
5	Right hemicolon	No	No	PCR	No	No

*MMR* mismatch repair, *dMMR* deficient of mismatch repair, *MSI-H* microsatellite instability-high

### Treatment and curative effect

Twenty-five locally advanced colon cancer patients underwent radical surgery. Three received neoadjuvant treatment, with a FOLFOX (5-fluorouracil, oxaliplatin plus leucovorin) regimen in 2 patients and neoadjuvant radiotherapy in one patient. Downstaging was not observed in these three patients, but all of them received adjuvant chemotherapy with FOLFOX regimen after surgery. Twenty patients did not receive neoadjuvant treatment, only adjuvant chemotherapy, with 12 patients receiving CAPOX (capecitabine plus oxaliplatin) and 8 patients receiving FOLFOX. The remaining two patients underwent surgery only.

In 11 locally advanced rectal cancer patients, only 4 (36.4%) received neoadjuvant therapy. One received CAPOX with Avastin combined with radiotherapy as part of a clinical trial, and he experienced pathological complete remission (pCR) after surgery. One received CAPOX combined with radiotherapy, and another received FOLFOX chemotherapy only. The pathology of these latter two patients after surgery suggested T and N downstaging. The last patient received CAPOX combined with radiotherapy. However, her tumor did not exhibit obvious regression and could not be completely removed, so palliative colostomy was performed.

dMMR and MSI-H were confirmed after one cycle of capecitabine oral administration for a distal T3N1M0 rectal cancer patient, demonstrating that immunotherapy alone was applied in her case. Pembrolizumab was given for six cycles with the addition of Ipilimumab in the second and third cycles. Clinical complete response (cCR) of the tumor was found after the sixth cycle of immunotherapy. A watch and wait strategy was suggested for her, and Pembrolizumab was prescribed continuously for another four cycles.

For the 33 patients who presented with distant metastasis, 5 patients did not receive any anti-cancer treatment, and another 3 patients had palliative chemotherapy only. The remaining 25 patients underwent surgery, with 10 having radical resection of primary and metastatic foci, and 15 underwent palliative surgery. Among these 25 patients, 4 received preoperative chemotherapy, 15 received postoperative chemotherapy, 1 received adjuvant radiotherapy, and 1 received chemotherapy both before and after surgery.

In summary, 44 patients underwent radical surgery and 16 patients underwent palliative surgery. Fifty-six patients received chemotherapy, and 6 patients received radiotherapy.

### Survival analysis

**Fig. 1:**
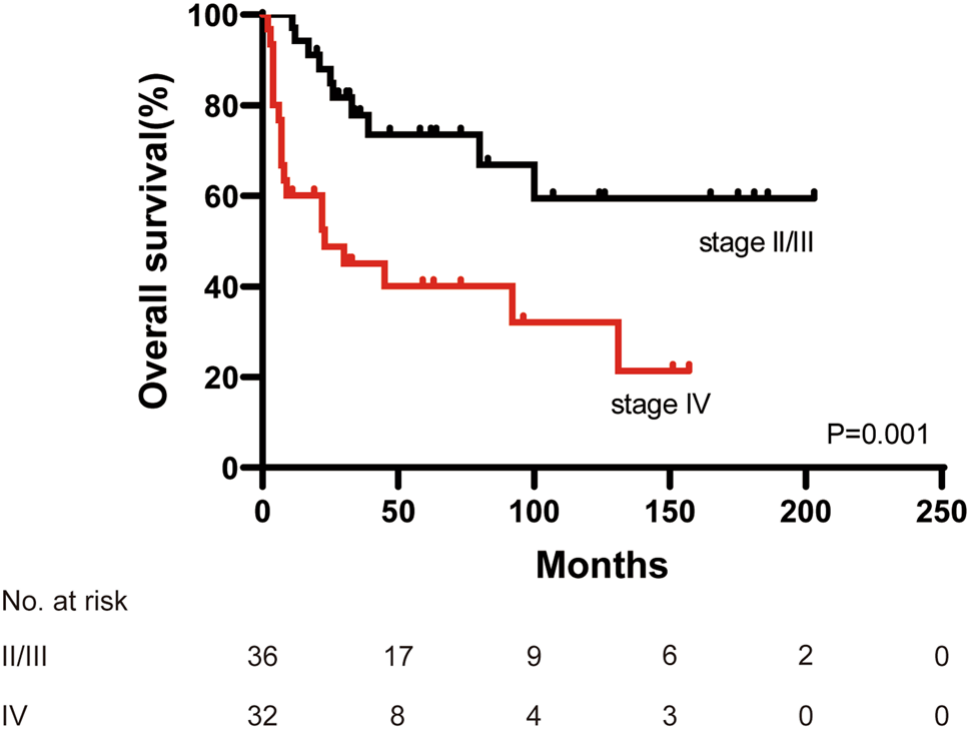
Kaplan–Meier survival curve of overall survival comparing stage II/III and IV (*p* = 0.001, log-rank test)

The median follow-up time of patients was 31 (0–203) months. Among all 70 patients, 4 (3.3%) were lost to follow-up. The median OS was 80.0 months for all patients. The 3-year and 5-year OS rates were 61.8% and 57.2%, respectively. When we investigated the association between clinical factors and outcomes using univariate analysis, only stage II/III (*p* = 0.001) was significantly associated with favorable survival (Table 3, Fig. 1).

**Table 3:** Univariate analysis for survival of all patients (*n* = 70)

Variables	*n*	mOS (month)	3-year OS rate (%)	5-year OS rate (%)	*p* value
Gender					0.332
Male	42	92.8	58.8	58.8	
Female	28	NA	66.6	54.5	
Primary tumor site^a^					0.096
Colon	50	131.1	67.8	61.4	
Rectum	19	80.0	50.2	50.2	
Pathological type					0.116
MA and SRCC	32	45.5	56.8	47.3	
Classic adenocarcinoma	38	NA	66.4	66.4	
Lymph node status					0.332
Positive	41	92.8	68.2	60.7	
Negative	10	NA	90.0	90.0	
Stage^b^					0.001
II/III	36	NA	77.8	73.5	
IV	33	23.4	44.7	39.7	
CEA^c^					0.452
≤ 5 ng/ml	17	NA	66.6	55.5	
> 5 ng/ml	29	80.0	58.3	58.3	
CA19-9^d^					0.863
≤ 35 U/ml	27	NA	59.5	52.0	
> 35 U/ml	19	131.1	64.7	64.7	
Period of diagnosis					0.502
2001–2009	28	45.5	53.8	50.0	
2010–2019	42	80.0	68.8	63.5	

*mOS* median overall survival, *NA* not available, *MA* mucinous adenocarcinoma, *SRCC* signet ring cell carcinoma, *CEA* carcinoembryonic antigen, *CA19-9* carbohydrate antigen 19–9

^a^Primary tumor location in one patient was unclear

^b^Staging in one patient was unclear

^c^Data of 46 patients were available

^d^Data of 46 patients were available

We then explored factors affecting the survival of locally advanced and distant metastatic CRCs.

**Fig. 2:**
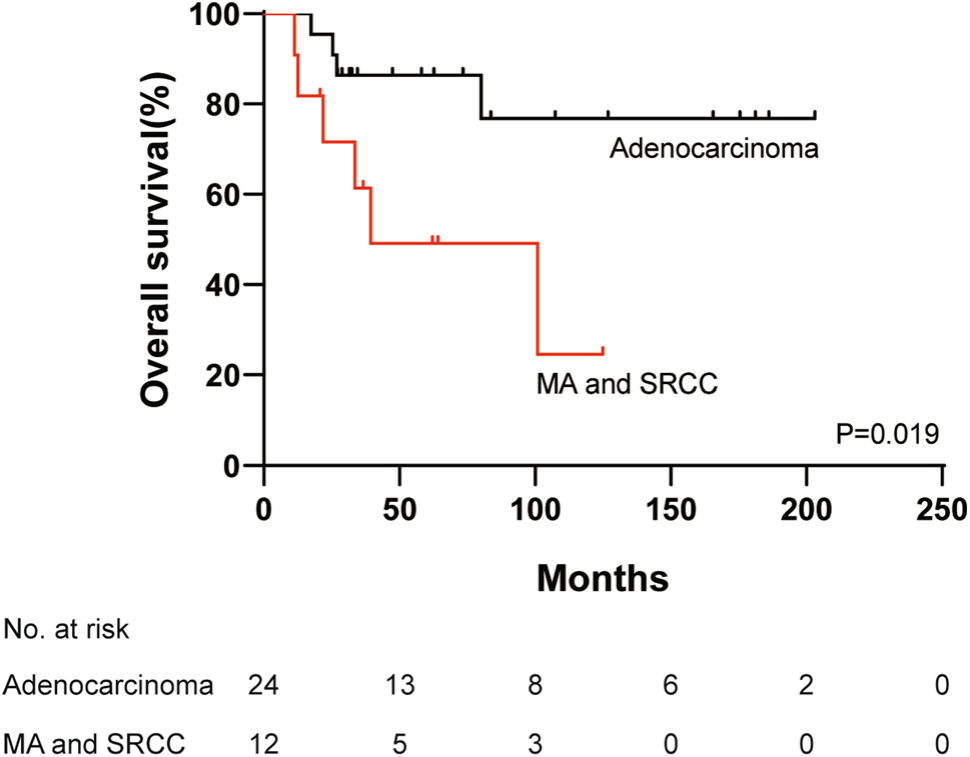
Kaplan–Meier survival curve of overall survival comparing classic adenocarcinoma vs. mucinous adenocarcinoma (MA) and signet ring cell carcinoma (SRCC) in stage II/III colorectal cancer patients (*p* = 0.019, log-rank test)

The median OS did not reach for locally advanced CRCs. The 3-year and 5-year OS rates were 77.8% and 73.5%, respectively. Classic adenocarcinoma (*p* = 0.019) (Table 4, Fig. 2) and radical surgery (*p* = 0.025) (Table 4) were significantly associated with favorable OS in univariate analysis. The 3-year and 5-year OS rates of classic adenocarcinoma were 86.4% and 86.4%, respectively, and the 3-year and 5-year OS rates of radical surgery were 80% and 75.6%, respectively. When the aforementioned factors were compared by a multivariate model using Cox proportional hazards regression analysis, radical surgery had the lower hazard ratio (HR) of 0.042 (95% CI 0.003–0.584; *p* = 0.018), and classic adenocarcinoma had an HR of 0.182 (95% CI 0.045–0.734; *p* = 0.017) (Table 4).

**Table 4:** Univariate and multivariate analysis for survival of locally advanced colorectal cancer patients (*n* = 36)

Variables	*n*	mOS (month)	3-year OS rate (%)	5-year OS rate (%)	Univariate analysis *p* value	Multivariate analysis		
						HR	95% CI	*p* value
Gender					0.329	NA		
Male	17	100.9	73.3	73.3				
Female	19	NA	81.2	73.0				
Primary tumor site					0.495	NA		
Colon	25	NA	77.4	71.5				
Rectum	11	100.9	78.8	78.8				
Pathological type					0.019			0.017
MA and SRCC	12	39.4	61.4	49.1		1		
Classic adenocarcinoma	24	NA	86.4	86.4		0.182	(0.045, 0.734)	
Stage^a^					0.270	NA		
II	9	NA	100.0	100.0				
III	25	NA	73.3	67.7				
Radical surgery					0.025			0.018
No	2	17.4	0	0		1		
Yes	34	NA	80.0	75.6		0.042	(0.003, 0.584)	
CEA^b^					0.206	NA		
≤ 5 ng/ml	8	NA	75.0	75.0				
> 5 ng/ml	11	NA	100.0	100.0				
CA19-9^c^					0.434	NA		
≤ 35 U/ml	12	NA	82.5	82.5				
> 35 U/ml	7	NA	100.0	100.0				

*mOS* median overall survival, *HR* hazard ratio, *NA* not available, *CI* confidence interval, *MA* mucinous adenocarcinoma, *SRCC* signet ring cell carcinoma, *CEA* carcinoembryonic antigen, *CA19-9* carbohydrate antigen 19–9

^a^Stage in two patients was unclear

^b^Data of 19 patients were available

^c^Data of 19 patients were available

**Fig. 3:**
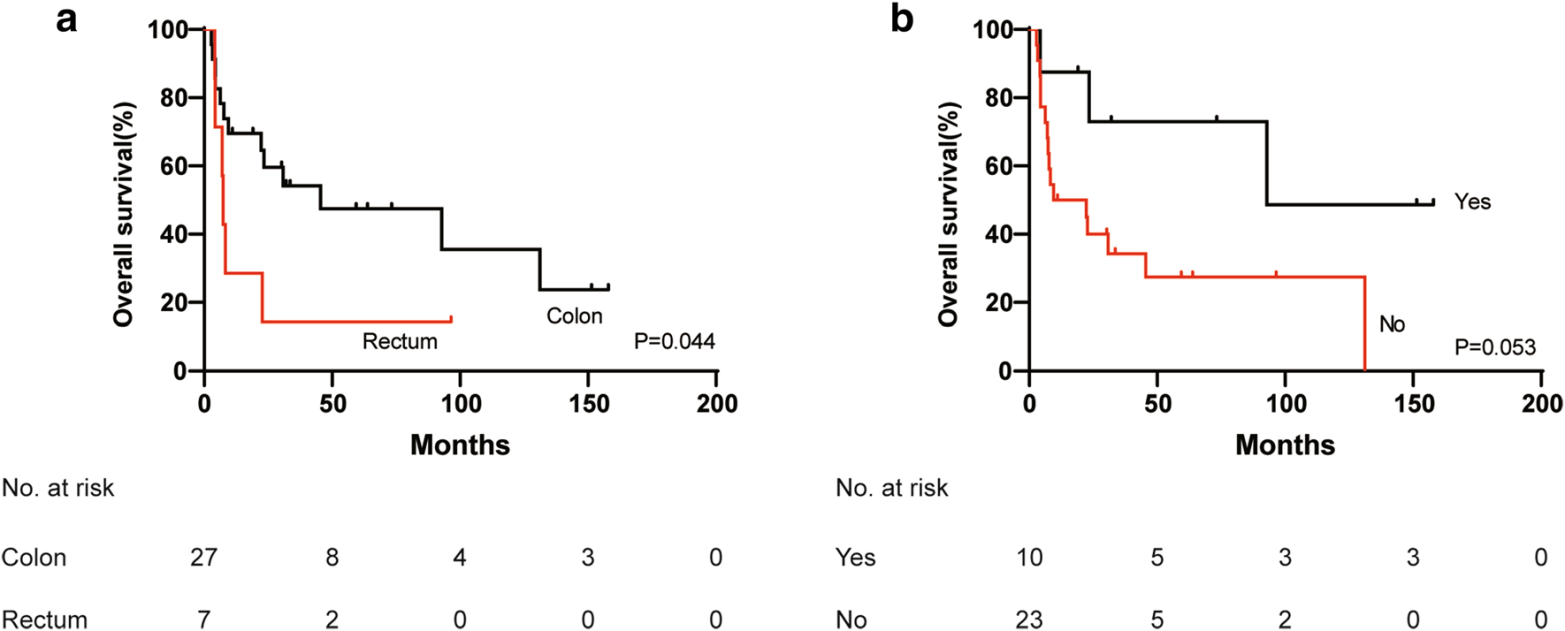
Kaplan–Meier survival curve of overall survival comparing rectum vs. colon in stage IV colorectal cancer patients (3**a**) (*p* = 0.044, log-rank test). Radical surgery vs. no radical surgery in stage IV colorectal cancer patients (3**b**) (*p* = 0.053, log-rank test)

With respect to distant metastatic CRCs, the median OS was 23.4 months, and the 3-year and 5-year OS rates were 44.7% and 39.7%, respectively. Only primary tumor location in the colon was significantly associated with favorable survival (*p* = 0.044) (Table 5, Fig. 3a). The 3-year and 5-year OS rates of colon cancer were 56.7% and 49.6%, respectively. The median OS in patients who underwent radical surgery was much longer than that in patients who did not (92.83 months vs. 9.5 months), although the *p* value was not significant (*p* = 0.053) (Table 5, Fig. 3b).

**Table 5:** Univariate analysis for survival of distant metastatic colorectal cancer patients (*n* = 33)

Variables	*n*	mOS (month)	3-year OS rate (%)	5-year OS rate (%)	*p* value
Gender					0.226
Male	24	92.8	50.2	50.2	
Female	9	22.7	28.6	0	
Primary tumor site^a^					0.044
Colon	25	45.5	56.7	49.6	
Rectum	7	7.4	14.3	14.3	
Pathological type					0.193
MA and SRCC	19	45.5	57.4	49.2	
Classic adenocarcinoma	14	7.4	28.8	28.8	
Radical surgery					0.053
Yes	10	92.8	72.9	72.9	
No	23	9.5	34.3	27.4	
CEA^b^					0.199
≤ 5 ng/ml	9	45.5	60.0	40.0	
> 5 ng/ml	17	7.4	31.1	31.1	
CA19-9^c^					0.954
≤ 35 U/ml	14	30.8	43.1	28.7	
> 35 U/ml	12	22.3	40.0	40.0	

*mOS* median overall survival, *NR* not reached, *MA* mucinous adenocarcinoma, *SRCC* signet ring cell carcinoma, *CEA* carcinoembryonic antigen, *CA19-9* carbohydrate antigen 19–9

^a^Primary tumor location in one patient was unclear

^b^Data of 26 patients were available

^c^Data of 26 patients were available

## Discussion

CRC rarely occurs in children and adolescents who are less than 20 years old. There are some case reports and small cohorts of child and adolescent CRCs (Shankar et al. 1999; Andersson et al. 1976; Ahn et al. 2017; Yang et al. 2015; Koh et al. 2015; Yeh et al. 2017; Vastyan et al. 2001; Noh et al. 2013), but there were only three cohorts (Sultan et al. 2010; Yang et al. 2010; Hill et al. 2007) with more than 50 patients found on PubMed, two of which were from the Surveillance, Epidemiology, and End Results (SEER) database. According to SEER statistics in the USA (Sultan et al. 2010), the age-adjusted incidence rate of CRC in children and adolescents was only 0.38 per million, and only 159 CRCs occurred in children and adolescents between January 1973 and December 2005. It is also rare in China. Of the 11,503 CRCs in the Cancer Hospital, Chinese Academy of Medical Sciences from January 1999 to December 2009, there were only 19 CRCs in patients who were between 10 and 20 years old (Du et al. 2015). No large cohort of child and adolescent CRCs has been previously reported from China. We only identified 70 patients under 20 from September 2000 to July 2019 in three hospitals. The present study is one of the largest cohorts to examine the clinicopathological and prognostic features of this disease.

The latest CRC statistics show that distant-stage diagnoses in patients younger than 50 years, 50–64 years, and 65 years and older are approximately 26%, 23%, and 19%, respectively (Siegel et al. 2020). However, nearly half of patients were stage IV in the present cohort. Presenting signs and symptoms in CRC are often vague and nonspecific, and no routine screening has been suggested. Child and adolescent CRC may have severe delays in diagnosis and/or possess intrinsically more aggressive behavior. Therefore, additional effort is needed for early recognition of this malignancy.

In previous reports, histological types of MA and SRCC are approximately 10% and 1% among all CRCs (Verhulst et al. 2012; Gopalan et al. 2011; Nitsche et al. 2013; Hyngstrom et al. 2012), but the proportions of MA and SRCC are higher in children and adolescents than in adults (Kaplan et al. 2019). In the current study, MA (22.9%) and SRCC (22.9%) histology was observed much more frequently than reported in adults. Several studies have indicated poor prognosis in patients with mucinous histology (Verhulst et al. 2012; Hyngstrom et al. 2012; Sung et al. 2008). However, other studies found that neither MA nor SRCC was an independent predictor of decreased survival (Li et al. 2019; Nitsche et al. 2016). Herein, we found that MA and SRCC indicated poor prognosis for LACRC.

Due to the rarity of CRC in patients from such young age groups, clinical management and treatment approaches are generally decided according to experiences from the management of adult patients. The NCCN and ESMO guidelines both recommend multimodal treatment of locally advanced rectal cancer (LARC), involving neoadjuvant concurrent fluoropyrimidine-based chemotherapy with pelvic radiation, total mesorectal excision (TME), and adjuvant chemotherapy. In our study, among the nine LARC patients who underwent radical surgery, only three received neoadjuvant chemoradiation. Infertility might be the primary reason for omitting radiotherapy in LARC patients. Radical surgery was not successfully performed in two patients, both of whom died within 2 years after diagnosis.

For metastatic patients, surgery and chemotherapy were the most mainstream treatments in the present study and five patients did not receive any anti-cancer treatment for some reason. Radical surgery, which occurred in approximately 33.3% of patients in our cohort, may represent the only method to prolong survival for them. A previously study reported 5-year survival rates in patients diagnosed with distant metastasis from the ages of 20–49, 50–64, and 65 and older of 21%,16%, and 10%, respectively (Siegel et al. 2020). In our study, the 5-year OS rate of CRCs in patients under 20 was 39.7%, which is much higher than in patients older than 20. The higher rate of radical surgery may contribute to this increased rate of survival.

Health providers should do further investigations, such as genetic studies, to better understand the disease and to identify age-appropriate solutions. Clinicians and patients should be more engaged because more drugs are currently available, such as molecular targeted drugs and a multidisciplinary therapy model, which is implemented in most hospitals.

dMMR was represented in 15–20% of stage II/III CRCs and in approximately 5% in the metastatic setting (Auclin et al. 2017). Five (23.8%) patients were dMMR, and 4 (66.7%) patients were MSI-H in our cohort, which is far higher than rates observed in adults. As dMMR/MSI-H is a major risk factor for CRC and involves a high percentage of dMMR/MSI-H in children and adolescent CRC patients, it is necessary to assess dMMR/MSI-H status when children and adolescents are diagnosed with CRC. However, MSI/MMR status was only available in a small number of patients, and more patients are needed for further validation. There was also a discrepancy between the results of immunohistochemistry and molecular detection in one patient who was dMMR but MSS and in one patient who was pMMR but MSI-H in our cohort. There are some reasons for this discrepancy. Some MMR gene variants do not affect the structure of their corresponding antigens and allow for retained normal MMR protein expression, but MMR proteins cannot recognize and repair DNA damage. Deficiencies in certain MMR proteins, such as MSH6, are not sufficient to result in MSI. Assessment of MSI and dMMR status discrepancy can be associated with a false-positive or a false-negative. Immune checkpoint blockades (ICBs), such as anti-PD-1 antibody and anti-CTLA-4 antibody, are effective in MSI-H/dMMR metastatic CRC. Recent studies in the refractory CRC setting have led to US Food and Drug Administration approvals for Pembrolizumab and Nivolumab (with or without Ipilimumab) for MSI-H/dMMR metastatic CRC.

In this study, the patient who received ICBs as neoadjuvant treatment achieved cCR and did not have surgery. Recently, PD-1 plus CTLA-4 blockade was demonstrated as highly effective in early-stage dMMR colon cancers (Chalabi et al. 2020). For the higher percentage of dMMR/MSI-H in child and adolescent CRCs, these patients may greatly benefit from immunotherapy. However, only a few patients have been reported (Chalabi et al. 2020; Zhang et al. 2019; Liu et al. 2020), and more data are needed for confirming and determining the best combination of immunotherapy with or without chemotherapy and/or radiotherapy to achieve a higher complete response rate and longer OS. There are some ongoing clinical trials attempting to add ICBs as neoadjuvant treatments for LACRC. In addition, we initiated a phase II clinical trial to combine PD-1 blockade with chemoradiation for dMMR/MSI-H unresectable CRCs. Genetic testing should be performed upon initial diagnosis for all child and adolescent CRCs, and immunotherapy is a promising modality for facilitating survival benefits in these patients.

There are some limitations to our study. This study is not a prospective cohort and has some of the inherent inadequacies of retrospective investigations. For example, molecular detections were available in only a small number of patients. In addition, the cohort covered nearly 20 years, thus a proportion of patients were lost, which may cause bias during analysis. Consequently, our observations warrant further consideration and validation in a larger patient series.

## Conclusions

Child and adolescent CRCs are prone to having distant metastasis, poor pathological type, left hemicolon location, and dMMR/MSI-H phenotype. Fortunately, active comprehensive treatment, including radical surgery, conveys a survival benefit. In addition, it is necessary to do a comprehensive examination in those who have symptoms as early as possible due to the prognosis of early-stage patients being much better.

## Data Availability

The data and material that support the findings of this study are available from the corresponding author upon reasonable request.

## Abbreviations

CRC: Colorectal cancer
dMMR: Deficient of mismatch repair
MSI-H: Microsatellite instability-high
OS: Overall survival
AYA: Adolescent and young adult
MA: Mucinous adenocarcinoma
SRCC: Signet ring cell carcinoma
AJCC: American Joint Committee on Cancer
TNM: Tumor-node-metastasis
CEA: Carcinoembryonic antigen
CA19-9: Carbohydrate antigen 19–9
MMR: Mismatch repair
IHC: Immunohistochemistry
NGS: Next-generation sequencing
PCR: Polymerase chain reaction
K-ras: Kirsten rat sarcoma viral oncogene
LACRC: Locally advanced colorectal cancer
MSS: Microsatellite stability
pMMR: Proficient of mismatch repair
pCR: Pathological complete remission
cCR: Clinical complete response
HR: Hazard ratio
CI: Confidence interval
SEER: Surveillance, Epidemiology, and End Results
NCCN: National Comprehensive Cancer Network
ESMO: European Society for Medical Oncology
LARC: Locally advanced rectal cancer
TME: Total mesorectal excision
ICB: Immune checkpoint blockade
